# Phase I clinical study of the recombinant antibody toxin scFv(FRP5)-ETA specific for the ErbB2/HER2 receptor in patients with advanced solid malignomas

**DOI:** 10.1186/bcr1264

**Published:** 2005-06-01

**Authors:** Gunter von Minckwitz, Sebastian Harder, Sascha Hövelmann, Elke Jäger, Salah-Eddin Al-Batran, Sibylle Loibl, Akin Atmaca, Christian Cimpoiasu, Antje Neumann, Aklil Abera, Alexander Knuth, Manfred Kaufmann, Dirk Jäger, Alexander B Maurer, Winfried S Wels

**Affiliations:** 1 Department of Gynecology and Obstetrics, University Hospital Frankfurt, Germany; 2 Institute for Clinical Pharmacology, University Hospital Frankfurt, Germany; 3 G2M Cancer Drugs AG, Frankfurt, Germany; 4 Medizinische Klinik II, Hämatologie-Onkologie, Krankenhaus Nordwest, Frankfurt, Germany; 5 Department of Oncology, University Hospital Zürich, Switzerland; 6 Chemotherapeutisches Forschungsinstitut Georg-Speyer-Haus, Frankfurt, Germany

## Abstract

**Introduction:**

ScFv(FRP5)-ETA is a recombinant antibody toxin with binding specificity for ErbB2 (HER2). It consists of an N-terminal single-chain antibody fragment (scFv), genetically linked to truncated *Pseudomonas *exotoxin A (ETA). Potent antitumoral activity of scFv(FRP5)-ETA against ErbB2-overexpressing tumor cells was previously demonstrated *in vitro *and in animal models. Here we report the first systemic application of scFv(FRP5)-ETA in human cancer patients.

**Methods:**

We have performed a phase I dose-finding study, with the objective to assess the maximum tolerated dose and the dose-limiting toxicity of intravenously injected scFv(FRP5)-ETA. Eighteen patients suffering from ErbB2-expressing metastatic breast cancers, prostate cancers, head and neck cancer, non small cell lung cancer, or transitional cell carcinoma were treated. Dose levels of 2, 4, 10, 12.5, and 20 μg/kg scFv(FRP5)-ETA were administered as five daily infusions each for two consecutive weeks.

**Results:**

No hematologic, renal, and/or cardiovascular toxicities were noted in any of the patients treated. However, transient elevation of liver enzymes was observed, and considered dose limiting, in one of six patients at the maximum tolerated dose of 12.5 μg/kg, and in two of three patients at 20 μg/kg. Fifteen minutes after injection, peak concentrations of more than 100 ng/ml scFv(FRP5)-ETA were obtained at a dose of 10 μg/kg, indicating that predicted therapeutic levels of the recombinant protein can be applied without inducing toxic side effects. Induction of antibodies against scFv(FRP5)-ETA was observed 8 days after initiation of therapy in 13 patients investigated, but only in five of these patients could neutralizing activity be detected. Two patients showed stable disease and in three patients clinical signs of activity in terms of signs and symptoms were observed (all treated at doses ≥ 10 μg/kg). Disease progression occurred in 11 of the patients.

**Conclusion:**

Our results demonstrate that systemic therapy with scFv(FRP5)-ETA can be safely administered up to a maximum tolerated dose of 12.5 μg/kg in patients with ErbB2-expressing tumors, justifying further clinical development.

## Introduction

Aberrant expression of the epidermal growth factor receptor or the closely related ErbB2 (HER2/neu) receptor tyrosine kinase has been implicated in the formation of various human malignancies [1,2], making these receptors interesting targets for directed anticancer therapeutics. Antibodies that block ligand binding or interfere with receptor function can directly inhibit the growth of cancer cells in addition to their potential to direct effector cells of the immune system to the tumor [3]. With the humanized mAb Herceptin™ (trastuzumab), an ErbB2-specific reagent for the treatment of breast carcinomas is in clinical use. Monotherapy with the antibody or combination with chemotherapy protocols resulted in increased clinical benefit for a significant proportion of patients with ErbB2-overexpressing metastatic breast cancers [4,5]. Nevertheless, responses could not be achieved in all patients with tumors expressing high ErbB2 levels, suggesting that in addition to enhanced expression of the target receptor, other factors such as limited recruitment of endogenous immune effector mechanisms or the presence of alternative signaling pathways in tumor cells can also influence treatment outcome.

In contrast to such unmodified antibodies, antibody toxins are not dependent on the inhibition of signaling or on the recruitment of complement or endogenous killer cells for antitumoral activity, but they combine antibody-mediated recognition of tumor cells with specific delivery of a potent cytotoxic effector molecule [6-8]. These tailor-made targeting reagents might therefore represent a valuable alternative to unmodified mAbs, and could complement their use in the clinic. ScFv(FRP5)-ETA is a recombinant single-chain antibody toxin with binding specificity for ErbB2-overexpressing tumor cells [9,10]. The N-terminal portion of the bacterially expressed molecule is contributed by a single-chain antibody fragment (scFv) derived from heavy-chain and light-chain variable domains of murine mAb FRP5, which recognizes the extracellular domain of human ErbB2 [11]. ScFv(FRP5)-ETA harbors a truncated *Pseudomonas aeruginosa *exotoxin A (ETA, PE) fragment (amino acids 252–613 of mature exotoxin A) at the C-terminus, which is devoid of the toxin's natural cell-binding domain [9]. Upon specific binding of the scFv domain to ErbB2 on the surface of tumor cells, the antibody toxin is internalized by receptor-mediated endocytosis, the enzymatic domain of the molecule is released into the cytoplasm and ADP ribosylates elongation factor 2, a critical component of the target cell's translation machinery [12]. Toxin-mediated inactivation of elongation factor 2 causes the inhibition of protein synthesis and results in subsequent tumor cell death by apoptosis [13,14].

Efficacy of scFv(FRP5)-ETA in the treatment of ErbB2-overexpressing tumors has been established in numerous preclinical *in vitro *and *in vivo *studies. ScFv(FRP5)-ETA displayed potent antitumoral activity *in vitro *against a wide range of established and primary human tumor cells, including breast and ovarian carcinomas [9,14,15], squamous cell carcinomas [10,16] and prostate carcinomas [17]. In experimental animals, locally or systemically applied scFv(FRP5)-ETA effectively inhibited the *in vivo *growth of human tumor xenografts [9,10,14,16], and murine and rat tumor cells stably transfected with human c-*erb*B2 constructs [18,19]. Thereby, complete elimination of subcutaneously growing tumors [16] and prevention of metastasis formation by disseminated cancer cells [19] was observed in some models.

Potent antitumoral activity in animal models has also been described for antibody toxins derived from the ErbB2-specific antibody e23 [20,21]. In cancer patients, however, intravenous application of such an e23-based antibody toxin resulted in severe liver toxicity, and effective doses could not be reached [22]. In contrast, for the scFv(FRP5)-ETA molecule utilizing the different FRP5 antibody domain, we could previously show that local treatment of cutaneous lesions of ErbB2-expressing tumors by intratumoral injection of the scFv(FRP5)-ETA molecule was well tolerated, and resulted in shrinkage or complete regression of injected tumor nodules in the majority of patients [23].

Here we now report the first systemic application of scFv(FRP5)-ETA in a phase I dose-finding study in human cancer patients, with the objective to assess the maximum tolerated dose and the dose-limiting toxicity. Furthermore, we have obtained data concerning pharmacokinetic properties of scFv(FRP5)-ETA and its ability to induce a neutralizing antibody response.

## Patients and methods

### Patients

Patients eligible for treatment with scFv(FRP5)-ETA had to be 18 years of age or older, with ErbB2-overexpressing tumors confirmed by immunohistochemistry (DAKO-Hercep test 3+) or fluorescence *in situ *hybridization analysis, showing clinical, radiological, or serological evidence for a progression. Other eligibility criteria included at least one previous palliative systemic chemotherapy treatment and an absence of any standard treatment option. Patients with serious illness or medical conditions besides the diagnosis of cancer, a Karnofsky index <60%, and immunoreactivity against scFv(FRP5)-ETA were excluded from the study. See Table 1 for further details on patient characteristics. The study was conducted in compliance with the Helsinki Declaration [24]. The treatment protocol and consent form were approved by the regulatory authorities and institutional ethics committees. Informed consent was obtained from the patients before therapy.

### ErbB2-specific antibody toxin

Recombinant scFv(FRP5)-ETA was produced as an experimental drug under Good Manufacturing Practice conditions and was kindly provided by Ciba Geigy AG (Basel, Switzerland). Bacterial expression and isolation of recombinant protein from inclusion bodies was carried out following a protocol adapted for large-scale production from the basic procedures described elsewhere [18]. Homogeneity of the material was analyzed by SDS-PAGE and Coomassie staining, and the identity of the purified protein was confirmed by immunoblot analysis and amino acid sequencing. The content of endotoxins (<10 EU/ml at 0.1 mg/ml protein), content of *Escherichia coli *proteins (<11 μg/ml) and content of DNA (<20 pg/ml) were determined following standard procedures (data not shown). The antibody toxin was supplied as a sterile solution at 0.3 mg/ml scFv(FRP5)-ETA in a phosphate buffer [23]. Aliquots of scFv(FRP5)-ETA solution were transferred to 2 ml vials under sterile conditions in the hospital pharmacy and were stored at -70°C. Upon thawing and subsequent storage at temperatures between 4°C and 8°C, scFv(FRP5)-ETA retained full bioactivity for a minimum of 6 days (IC_50 _for ErbB2-expressing Renca-lacZ/ErbB2 cells, 5–6 ng/ml) [19,23]. For application, the required amount of antibody toxin was thawed and kept until use for a maximum of 5 days at temperatures between 4°C and 8°C.

### Treatment schedule and dose levels

Experiments in mice and rats demonstrated efficacy of daily intravenous injections of scFv(FRP5)-ETA for 10 days against localized and metastatic tumors [18,19,25]. Based on these data, the treatment schedule for the phase I clinical study was developed: patients received a total of 10 intravenous infusions of scFv(FRP5)-ETA on day 1, day 2, day 3, day 4, day 5, day 8, day 9, day 10, day 11, and day 12. ScFv(FRP5)-ETA was diluted in physiological Ringer solution to achieve a total injection volume of 50 ml. A test dose of 10 μg intravenously was given as a 15-min infusion on day 1. The remaining dose was administered 4 hours later as a 15-min infusion. The dose levels of scFv(FRP5)-ETA were 2, 4, 10, 12.5, or 20 μg/kg per treatment day. Patients received 8 mg dexamethasone, 50 mg ranitidine, and 2 mg clemastine as a supportive treatment 30 min prior to scFv(FRP5)-ETA infusion to avoid anaphylactic reactions.

### Tumor assessments

The modified Response Evaluation Criteria in Solid Tumors were used for objective tumor response assessment in this trial. Chest X-ray, abdominal ultrasound or computed tomography scan, specific measurement of an indicator lesion, bone scan, or bone X-ray in the case of hot spots in the bone scan were performed within 3 months before therapy and on day 29 after the onset of scFv(FRP5)-ETA therapy. To ensure comparability, every effort was made to use the same instrumental examination from baseline through follow-up.

### Safety evaluation

The overall proportion of patients experiencing any toxicity was determined using the National Cancer Institute Common Toxicity Criteria, and the corresponding grading system was used to grade adverse events for recording in the case report form. For all adverse events not classified by National Cancer Institute Common Toxicity Criteria, the COSTART grading classification [26] was used (severity: 1, mild; 2, moderate; 3, severe; and 4, life-threatening). Cardiac function was determined before treatment and was monitored by electrocardiography, by Multiple Gated Acquisition scan, or by echocardiography on day 22 after initiation of therapy to assess potential cardiotoxicity.

### Detection of scFv(FRP5)-ETA plasma levels by sandwich ELISA

Ninety-six-well microtiter plates (Greiner Bio-One, Frickenhausen, Germany) were coated overnight with 100 μl/well of 9 μg/ml goat anti-exotoxin A capture antibody (List Biological Laboratories, Campbell, CA, USA) diluted in 50 mM carbonate buffer, pH 9.5. After washing with PBS, the plates were blocked for 2 hours with PBS containing 1% BSA. For standard, control or patients' samples, 100 μl/well serum diluted 1:10 with PBS containing 6 mM ethylenediamine tetraacetic acid were added in duplicate, and were incubated for 2.5 hours at 37°C. After washing with PBS, 100 μl rabbit anti-exotoxin A antibody [9], diluted 1:250 in PBS, were added to each well for 1 hour at 37°C. After another washing step, bound rabbit antibodies were detected with 100 μl/well horseradish peroxidase-coupled anti-rabbit IgG antibody (Amersham Biosciences, Freiburg, Germany) for 1 hour at 37°C, after a final wash followed by 100 μl/well of 1% 3,3',5,5'-tetramethyl-benzidine (Sigma-Aldrich, Deisenhofen, Germany) substrate solution for 5–15 min at room temperature. Then the reaction was stopped by adding 50 μl/well of 1 M HCl, and the absorbance at 450 nm was measured using a Wallac Victor 2 ELISA reader (PerkinElmer Wallac, Freiburg, Germany).

### Pharmacokinetic analysis

The main parameters measured were *C*_max _and the area under the concentration–time curve (AUC_0–5 hours_) of scFv(FRP5)-ETA at day 5, obtained from the individual concentration–time profiles (area under the concentration–time curve by trapezoidal rule) and using the software program TOPFIT^® ^2.0 (Fischer Verlag, Stuttgart, Germany). Furthermore, the elimination half-life and plasma clearance were determined from the concentration–time profiles using a noncompartmental approach from the TOPFIT Library.

### Detection of circulating antibodies

Ninety-six-well microtiter plates (Greiner Bio-One) were coated overnight with 100 μl/well of 1 μg/ml scFv(FRP5)-ETA in PBS. After washing with PBS, the plates were blocked for 2 hours with PBS containing 1% BSA. For standard, control or patients' samples, 100 μl/well serum diluted 1:50 with PBS were added in duplicate, and were incubated for 3 hours at 37°C. After washing with Tris-buffered saline, 100 μl alkaline phosphatase-coupled rabbit anti-human IgG/IgM antibody (Sigma-Aldrich), diluted 1:2000 in PBS, were added to each well for 1 hour at 37°C, after a final wash followed by 100 μl/well nitrophenylpyrophosphate substrate solution at room temperature. The absorbance at 450 nm was measured using a Wallac Victor 2 ELISA reader (PerkinElmer Wallac). Neutralizing antibodies were determined in cell viability assays as described previously [23].

## Results

The primary objectives of the study were the determination of the maximum tolerated dose and the dose-limiting toxicity (grade 4 hematologic toxicity or grade 3 nonhematologic toxicity) of scFv(FRP5)-ETA after intravenous application. The secondary objectives were the determination of the pharmacokinetic profile of scFv(FRP5)-ETA, the time to progression, the objective response rate (complete and partial), and the immunological response to treatment.

Eighteen patients suffering from ErbB2-expressing metastatic breast cancers (13 patients), prostate cancers (two patients), head and neck cancer (one patient), non small cell lung cancer (one patient), or transitional cell carcinoma (one patient) (Table 1) were given at least five daily infusions of scFv(FRP5)-ETA, with total daily doses ranging from 100 μg to 1.4 mg. A total of 15 patients received the complete treatment cycle of 10 days without showing signs of dose-limiting toxicity, whereas therapy in three patients was stopped on day 8 due to a grade 3–4 elevation of liver enzymes (alanine aminotransferase [ALT], aspartate aminotransferase [AST]) (Table 2). A dose escalation scheme was pursued, which started at 2 μg/kg scFv(FRP5)-ETA per treatment day, followed by 4, 10, and 20 μg/kg scFv(FRP5)-ETA. Due to dose-limiting toxicity in two out of three patients treated at 20 μg/kg scFv(FRP5)-ETA, a protocol amendment was adopted to include a further dose level at 12.5 μg/kg scFv(FRP5)-ETA. Five out of six patients at 12.5 μg/kg scFv(FRP5)-ETA received the complete dose without experiencing severe side effects. In one patient at this dose level, however, therapy had to be stopped on day 8 due to toxicity. No objective response was observed in any of the patients, but two patients remained in stable disease for more than 3 months, and clinical signs of activity were seen in three patients.

### Toxicity

An increase in the serum levels of liver enzymes was found in the majority of the patients, with grade 1–2 elevation of ALT or AST seen in seven patients and grade 1–2 elevation of gamma-glutamyl transferase in six patients. A dose-limiting toxicity with grade 3–4 elevation of ALT or AST was found in two of three patients treated at 20 μg/kg scFv(FRP5)-ETA and in one patient treated at 12.5 μg/kg scFv(FRP5)-ETA, resulting in discontinuation of therapy in these patients on day 8. Enzyme levels returned to baseline values within 14 days after cessation of therapy in all patients. One patient with liver metastases, treated at the lowest dose level (2 μg/kg), developed cholestasis, which was due to massive disease progression but was unrelated to treatment, requiring a cessation of therapy on day 10. Another patient developed fever and dyspnoe on day 23 after onset of therapy, which resolved with antibiotics. However, this patient died on day 40, most probably unrelated to therapy but due to massive disease progression.

### Anti-tumoral efficacy

Complete or partial remissions were not observed after scFv(FRP5)-ETA treatment, which was not unexpected given the severity of the patients' disease and their tumor load. Stable disease was seen in two patients, however: one prostate cancer patient treated at 10 μg/kg scFv(FRP5)-ETA per day, and one breast cancer patient treated at 12.5 μg/kg scFv(FRP5)-ETA per day. Furthermore, clinical signs of activity were observed in another three patients, with two patients treated at 10 and 12.5 μg/kg scFv(FRP5)-ETA per day experiencing signs of healing of cancer-related cutaneous lesions. In the patient receiving 10 μg/kg scFv(FRP5)-ETA, a reduction in the size of a cervical lymph node metastasis was also observed and the morphine dose could be reduced by 50%. A third patient treated at 20 μg/kg scFv(FRP5)-ETA per day, despite discontinuation of treatment on day 8 due to dose-related side effects, demonstrated signs of an inflammatory response and softening of a large tumor mass in her right breast. Interestingly, the breast cancer patient with stable disease and two of the patients with clinical signs of activity had previously progressed under therapy with trastuzumab (Table 1).

### Pharmacokinetic profile

**Figure 1:**
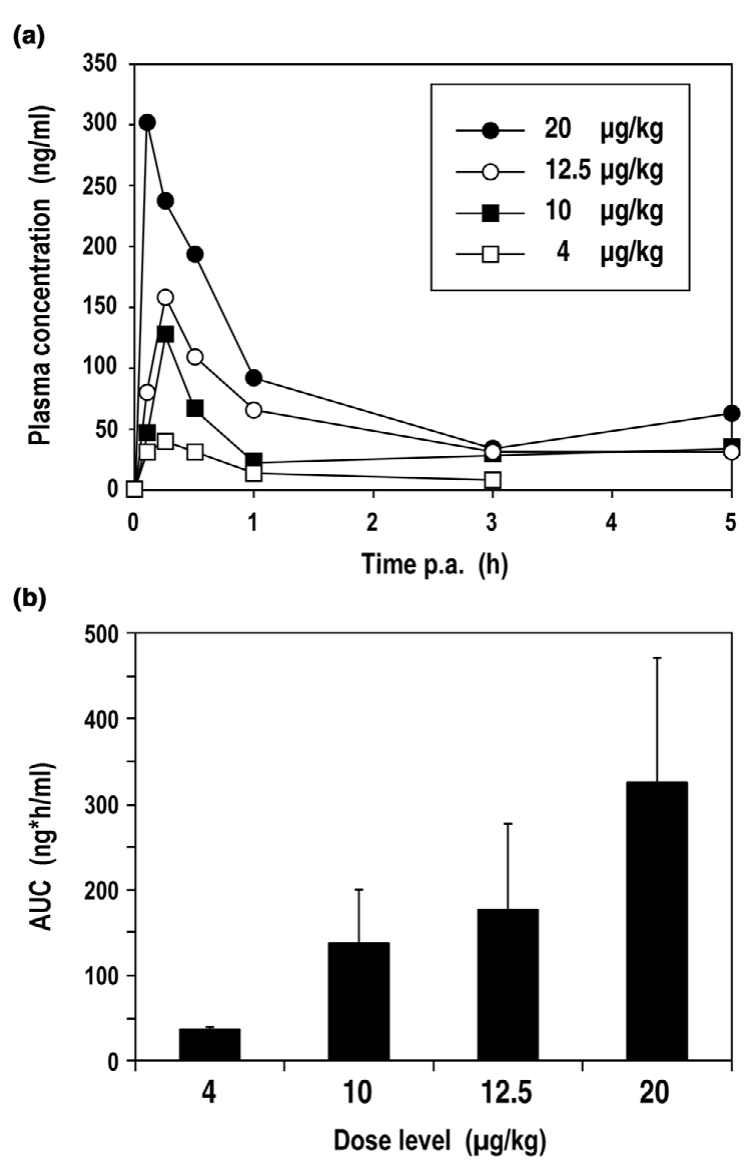
**(a) **Plasma levels of scFv(FRP5)-ETA at steady state. Plasma samples were taken from individual patients at each dose level at the indicated time points before and after infusion of scFv(FRP5)-ETA on treatment day 5. Plasma concentrations were determined by capture ELISA with 1:10 diluted plasma including standard scFv(FRP5)-ETA concentrations for quantification. No scFv(FRP5)-ETA was detected in the predose levels, indicating that no accumulation occurred with a once-daily dose interval. **(b) **Area under the concentration–time curve (AUC_0–5 hours_) at steady state on treatment day 5 for the different dose levels. The area under the concentration–time curve was calculated according to the trapezoidal rule from 0 to 5 hours. In accordance with the concentration-time profiles there was a dose-dependent increase of the area under the concentration–time curve, indicating linear pharmacokinetic behavior in the investigated dose range.

**Figure 2:**
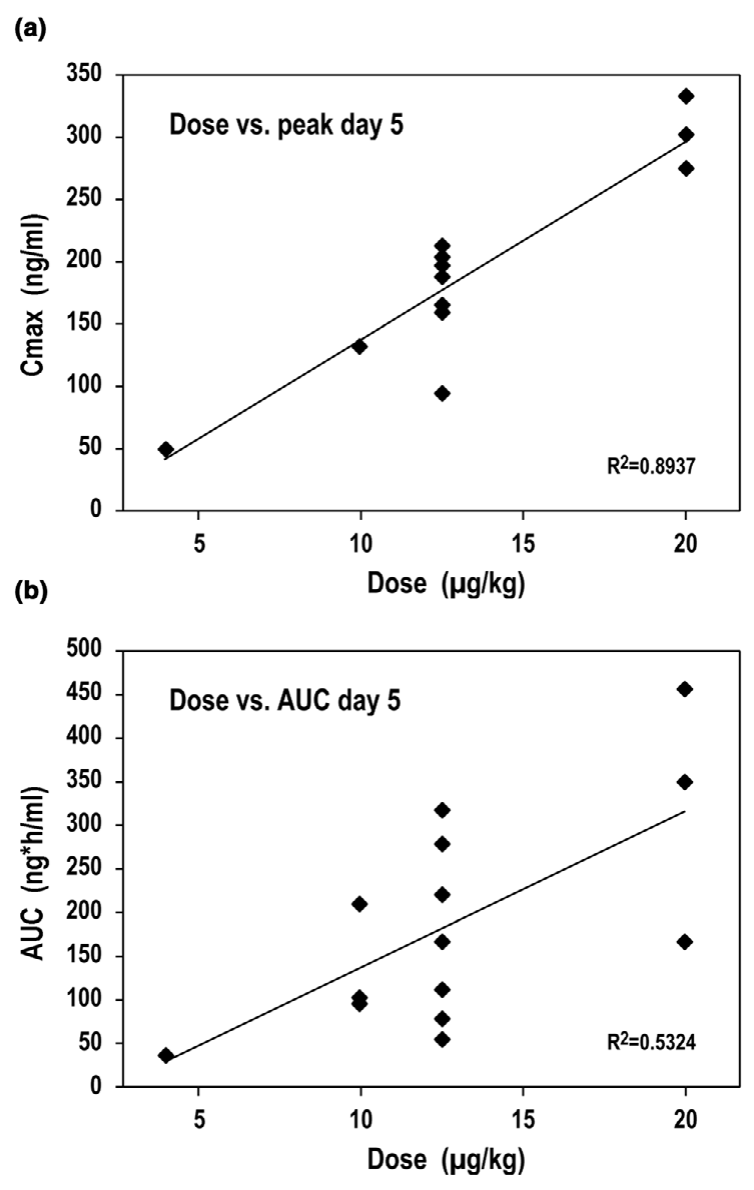
Pharmacokinetic parameters for individual patients on treatment day 5. **(a) **Dose versus peak plasma concentration, *C*_max_. **(b) **Dose versus area under the concentration–time curve (AUC).

No scFv(FRP5)-ETA could be detected in patient plasma at the 2 μg/kg dose. At dose levels equal to or greater than 4 μg/kg scFv(FRP5)-ETA per day, there was a good correlation between the dose level and the plasma concentration, with peak levels of scFv(FRP5)-ETA reached 15 min after the beginning of infusion, and a fast decline to baseline levels within 4 hours, indicating that the drug was not accumulating (Fig. 1a). Peak concentrations at steady state (day 5 of therapy) ranged from 18 to 49 ng/ml (mean, 39 ng/ml) at 4 μg/kg scFv(FRP5)-ETA, ranged from 128 to 129 ng/ml (mean, 129 ng/ml) at 10 μg/kg scFv(FRP5)-ETA, ranged from 93 to 204 ng/ml (mean, 160 ng/ml) at 12.5 μg/kg scFv(FRP5)-ETA, and ranged from 115 to 307 ng/ml (mean, 236 ng/ml) at 20 μg/kg scFv(FRP5)-ETA (Fig. 2a). The correlation between the dose of scFv(FRP5)-ETA and the area under the concentration–time curve was less pronounced (Figs 1b and 2b). Plasma clearance was calculated as 6.6 l/hour at 4 μg/kg, as 5.3 l/hour at 10 μg/kg, as 4.9 l/hour at 12.5 μg/kg, and as 3.8 l/hour at 20 μg/kg. The calculated half-life of scFv(FRP5)-ETA at the three higher dose levels was approximately 44 min. The pharmacokinetic data are summarized in Table 3.

### Immunogenicity

**Figure 3:**
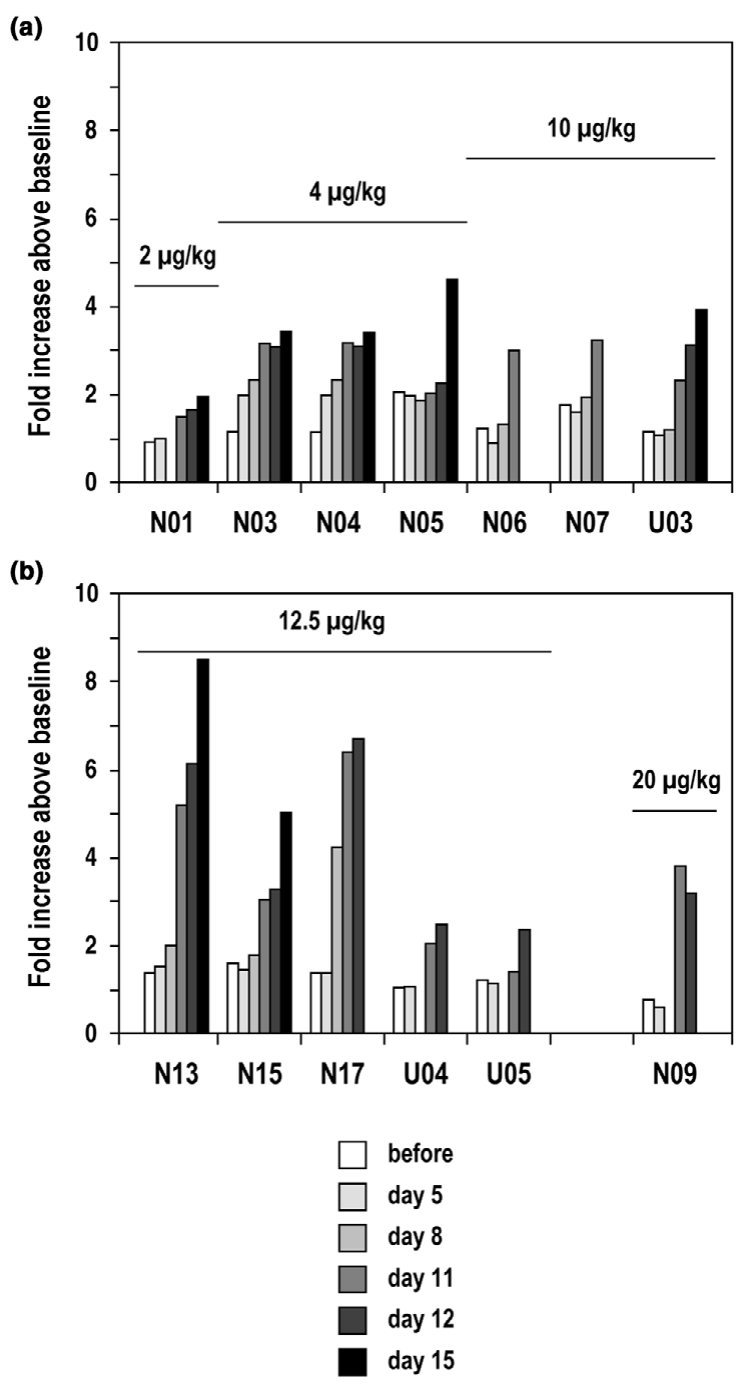
Development of scFv(FRP5)-ETA-specific antibodies after treatment. Relative levels of scFv(FRP5)-ETA-specific antibodies induced in patients by the treatment were determined by ELISA with plates coated with the antibody toxin. Sera taken on day 0 before treatment, and sera taken at the indicated days after onset of therapy were diluted 1:50 for analysis. The baseline was determined using several human control sera negative for scFv(FRP5)-ETA-specific antibodies (not shown). Dose levels and patients are indicated.

Antibody responses to scFv(FRP5)-ETA were analyzed in detail in 13 patients. None of the patients had pre-existing antibodies reactive with scFv(FRP5)-ETA, but most patients developed antibodies to scFv(FRP5)-ETA beginning on day 8 after onset of therapy, as determined by ELISA (Fig. 3). Only in five patients, however, could antibodies with neutralizing capacity against scFv(FRP5)-ETA be detected in cell viability assays. A strong neutralizing capacity (neutralizing activity at a serum dilution of 1:100) was only seen in two patients, whereas another three patients developed weak or moderate neutralizing activity (neutralizing activity only at a serum dilution of 1:50) (data not shown). Clinical symptoms were not associated with these responses (Table 2).

## Discussion

Previous experimental work in various rodent models provided evidence for efficacy of the recombinant antibody toxin scFv(FRP5)-ETA against ErbB2-expressing tumors [9,10,14,16,18,19]. Further support for the use of scFv(FRP5)-ETA in tumor therapy arose from a study investigating intratumoral injection of the antibody toxin in patients with dermal metastases of ErbB2-expressing tumors [23]. Here we report results on the first systemic application of scFv(FRP5)-ETA in cancer patients, demonstrating safety at doses up to 12.5 μg/kg per day.

The predominant dose-limiting toxicity encountered in two of three patients treated at 20 μg/kg was transient liver toxicity, as indicated by a grade 3–4 elevation of serum liver enzymes (patient N10, ALT grade 4, AST grade 3; patient N12, ALT grade 3). An amendment to the protocol was implemented to test a dose level of 12.5 μg/kg. Liver toxicity (elevation of ALT/AST grade 3) was observed at this amended dose level in only one out of six patients. Therefore, 12.5 μg/kg was determined as the maximum tolerated dose. Liver toxicity can be a common complication encountered after treatment with recombinant toxins based on ETA. This was drastically demonstrated in a clinical study using erb-38, an ErbB2-specific toxin similar to scFv(FRP5)-ETA, which consists of a disulfide-bridged Fv fragment of the ErbB2-specific monoclonal antibody e23 linked to truncated *Pseudomonas *toxin [21]. Intravenous injection of 1 or 2 μg/kg recombinant protein every other day caused liver toxicity in all patients after 3 days of treatment [22]. This approximately 10-fold difference in the daily doses causing liver toxicity when comparing erb-38 with scFv(FRP5)-ETA might be explained by direct adverse effects of erb-38 against hepatocytes due to low ErbB2 expression in the liver, as postulated by Pai-Scherf and colleagues [22].

In tissue culture, scFv(FRP5)-ETA displayed selective cytotoxicity towards ErbB2-overexpressing tumor cells with IC_50 _values in the nanograms per milliliter range [9,17]. While scFv(FRP5)-ETA and other ErbB2-specific antibody toxins have not been compared directly, the same cell lines and similar assays were used in some studies for *in vitro *characterization. For example, when tested in protein synthesis inhibition assays, scFv(FRP5)-ETA displayed an IC_50 _value towards SKBR3 breast carcinoma cells of 29 ng/ml [9], compared with a value of 32 ng/ml for e23(Fv)PE40, an ErbB2-specific molecule employing a scFv antibody fragment of e23 for cell targeting [20]. Improved cytotoxic activity was reported for erb-38 [21], which might indeed explain the higher degree of toxicity of this antibody toxin in cancer patients. It is noteworthy, however, that significantly lower toxicity of scFv(FRP5)-ETA was also seen in animal experiments, where specific binding to endogenous ErbB2 on liver cells can be excluded as the cause for toxicity. ScFv(FRP5)-ETA could be applied intravenously in mice at doses up to 1 mg/kg daily for 10 days without causing any measurable side effects [19]. This contrasts with erb-38, for which an LD_50 _value in mice of 450 μg/kg was reported after three doses given every other day, and which caused death of animals by hepatic failure [22].

Various studies link the hepatotoxic effects of *Pseudomonas *exotoxin to the increased production of tumor necrosis factor alpha (TNF-α) by Kupffer cells in the liver and the resulting liver damage from activated T cells [27]. Mice depleted of T cells prior to ETA challenge failed to develop acute hepatic failure, whereas mice not immunologically compromised demonstrated hepatocyte apoptosis and increased plasma transaminase activity. Furthermore, in mice treated with the ETA-containing antibody toxin LMB-2, inhibition of TNF-α production in Kupffer cells by a specific TNF-binding protein or indomethacin prevented LMB-2-induced liver damage [28]. Agents such as infliximab, which neutralize the effects of TNF-α, are currently in clinical use for the treatment of rheumatoid arthritis and Crohn's disease [29]. These substances as well as nonsteroidal anti-inflammatory drugs may also be of use in preventing some of the unspecific toxic effects of ETA-based antibody toxins in humans.

Generally, cross-reactivity with normal tissues and severity of adverse reactions might at least in part depend also on the nature and the position of the epitope recognized by the antibody domain [30]. The mAb FRP5 and its scFv derivatives recognize a peptide epitope located in the N-terminal region of the receptor [14,31]. In contrast, the humanized ErbB2-specific antibody Herceptin™ (trastuzumab), which can induce cardiotoxicity in some patients [32], recognizes the juxtamembrane region of ErbB2 [33]. As in a previous study with locally applied scFv(FRP5)-ETA [23], here we have not observed cardiovascular complications in any of the patients treated with scFv(FRP5)-ETA, nor were such toxicities reported for the erb-38 molecule based on mAb e23 [22], for which information on the binding epitope is not available.

Our results demonstrate that intravenous administration of scFv(FRP5)-ETA leads to serum concentrations of the recombinant protein over several hours, predicted to be therapeutically relevant. IC_50 _values ranging from 10 to 100 ng/ml were determined in *in vitro *experiments with human tumor cells [16]. In the present study a peak serum concentration of 129 ng/ml was found in cancer patients at a dose of 10 μg/kg scFv(FRP5)-ETA, and serum concentrations between 50 and 100 ng/ml could be maintained for 2 hours after administration of 12.5 μg/kg scFv(FRP5)-ETA. The calculated half-life of 44 min indicates that the recombinant toxin is rapidly cleared from the body and is not accumulating. Nevertheless, systemic treatment with scFv(FRP5)-ETA in mice was successful despite a relatively short half-life of 30 min in the circulation [9], suggesting that sufficient amounts of the molecule could reach the tumor site.

Although objective responses could not be achieved in this study, clinical signs of activity such as healing of cutaneous lesions and stable disease were observed in some of the patients treated with scFv(FRP5)-ETA. The lack of major responses to treatment may be due to the advanced stage of the patients' disease, the limited treatment time of 2 × 5 days, or the limited number of patients treated at higher dose levels. Furthermore, problems with tumor cell accessibility can occur. Solid tumors of epithelial origin are often poorly vascularized, possibly limiting the use of large therapeutic molecules such as antibodies and antibody toxins when administered as a single agent. This might explain why most progress has been made so far in the clinical development of antibody toxins that target cell surface molecules such as CD22 and CD25 expressed on certain malignancies of hematologic origin [34,35], where tumor cells are usually more accessible. When scFv(FRP5)-ETA was directly administered into tumor lesions by intratumoral injection in a previous study, complete regression or partial reduction in the size of injected tumor nodules was found in five out of seven patients with tumors expressing high ErbB2 levels [23], indicating that the antibody toxin can very well selectively eliminate ErbB2-overexpressing target cells if they are accessible.

Antibody responses to scFv(FRP5)-ETA were analyzed in 13 patients. While none of these patients had pre-existing antibodies to ETA, they all developed antibody responses of varying intensity to scFv(FRP5)-ETA measurable after 8 days of therapy. Importantly, only in five of the 13 patients analyzed did these antibodies have scFv(FRP5)-ETA neutralizing activity, and two of these patients had stable disease for longer than 3 months. The pharmacokinetic parameters shown were determined on treatment day 5. Thereby a tendency towards a lower circulation half-life and lower area under the concentration–time curve values was found when compared with treatment day 1 (data not shown). This could at least partially be due to onset of anti-toxin antibody responses. During the second week of treatment, anti-toxin antibodies might have affected the half-life of scFv(FRP5)-ETA further, which was not formally investigated. Nevertheless, in a previous study, complete remission of tumor nodules locally injected with scFv(FRP5)-ETA could be achieved after a second treatment cycle despite pre-existing neutralizing antibodies induced during the first round of treatment [23]. Continued treatment of patients after development of anti-toxin antibodies might therefore still be efficacious.

## Conclusion

Taken together, our results demonstrate that the ErbB2-specific antibody toxin scFv(FRP5)-ETA can be safely administered intravenously at doses up to 12.5 μg/kg per day to treat cancer patients with ErbB2-overexpressing tumors. Thereby, serum concentrations of the drug were reached that could be of therapeutic value. The major dose-limiting side effect of scFv(FRP5)-ETA was hepatotoxicity. This may become controllable in future studies by using TNF neutralizing reagents or by temporary suppression of T-cell activation. Whether the development of neutralizing antibodies will limit the therapeutic utility of the antibody toxin or whether alternative treatment schedules or immunosupressant co-medication can overcome this problem remains unclear at present. To further investigate the efficacy of systemic scFv(FRP5)-ETA therapy in cancer patients, we recommend the dose of 12.5 μg/kg for subsequent, carefully planned phase II studies.

## Abbreviations

ALT = alanine aminotransferase; AST = aspartate aminotransferase; BSA = bovine serum albumin; ELISA = enzyme-linked immunosorbent assay; ETA = *Pseudomonas *exotoxin A; mAb = monoclonal antibody; PBS = phosphate-buffered saline; TNF-α = tumor necrosis factor alpha.

## Competing interests

G2M Cancer Drugs AG holds rights for commercial development of the study drug scFv(FRP5)-ETA. ABM, SHö and AAb have contributed to the study as employees of G2M AG. WSW is a shareholder of G2M AG. GvM, SHa, EJ, SEAB, SL, AAt, CC, AN, AK, MK and DJ declare that they have no competing interests.

## Authors' contributions

GvM, ABM, DJ, AK, MK, EJ, SHa and WSW participated in the design and coordination of the study. GvM, DJ, EJ, SEAB, SL, AAt, CC, AK and MK provided the clinical data. ABM, SHö, SHa, AAb and AN performed and evaluated the assays to determine pharmacokinetic parameters and antibody responses to scFv(FRP5)-ETA. WSW, ABM, SHö, SHa and GvM drafted the manuscript. All authors read and approved the final manuscript.

## Figures and Tables

**Table 1:** Patient characteristics

Patient	Age (years), sex	Cancer type	Stage at diagnosis/surgery	Sites of metastasis	Prior therapy
N01	61, female	Head and neck	T2N2bMX	Local, intrapulmonal, mediastinal	Surgery, radiation, chemotherapy
U01	61, female	Breast	T2N2M1	Liver, bone	Surgery, chemotherapy, herceptin
U02	56, female	Breast	T4N1bM0	Lymph node, liver, central nervous system, skin, bone	Surgery, radiation, chemotherapy, herceptin
U03	68, female	Breast	T3N1M0	Lymph node, bone	Surgery, radiation, chemotherapy, herceptin, hormonal therapy
U04	64, female	Breast	T3N0M0	Local, skin, bone, intrapulmonal	Surgery, chemotherapy, herceptin
U05	71, female	Breast	T1aN3cM1	Lymph node, skin	Surgery, chemotherapy, herceptin
N03	63, female	Breast	T3N1bM0	Liver	Surgery, chemotherapy, herceptin
N04	55, male	Transitional cell carcinoma	TXNXMX	Lymph node	Surgery, chemotherapy, herceptin
N05	72, male	Prostate	T4N3M1	Lymph node, bone, other	Surgery, hormonal therapy
N06	63, male	Prostate	T2bN0M0	Lymph node	Surgery, chemotherapy, hormonal therapy
N07	74, female	Breast	T1N0M0	Liver	Surgery, radiation, hormonal therapy
N09	50, female	Breast	T1N0M0	Lymph node, liver, bone, intrapulmonal, pleural	Surgery, chemotherapy
N10	45, female	Breast	T3bN1bMX	Bone	Surgery, radiation, chemotherapy, herceptin
N12	69, female	Breast	T4N3M1	Local, lymph node, skin	Chemotherapy, hormonal therapy, immunotherapy
N13	46, female	Breast	TXNXMX	Lymph node, liver, central nervous system, skin, bone	Surgery, radiation, chemotherapy, herceptin
N14	82, female	Breast	TXNXMX	Liver, pleural	Surgery, hormonal therapy
N15	70, female	Breast	T2N1MX	Lymph node, skin, bone	Surgery, radiation, chemotherapy, herceptin
N17	62, male	Non small cell lung carcinoma	T2cN2cM1	Lymph node, bone, intrapulmonal	Patient refused standard therapy

**Table 2:** Study summary

Patient	Dose level (μg/kg)	Course of therapy	Toxicities ≥ grade 1	Dose-limiting toxicity	Neutralizing antibodies	Clinical response
N01	2	According to plan	GGT grade 2	No	No	Progression
U01	2	According to plan	None	No	n.d.	Progression
U02	2	Stopped on day 10	Cholestasis due to liver metastasis^a^	No	n.d.	Progression
						
N03	4	According to plan	GGT grade 2	No	No	Progression
N04	4	According to plan	ALT grade 1	No	No	Progression
N05	4	According to plan	Hemoglobin grade 3^a^	No	No	Progression
						
N06	10	According to plan	ALT grade 2, AST grade 1	No	+	Stable disease
N07	10	According to plan	ALT/AST grade 1, GGT grade 2	No	No	Progression
U03	10	According to plan	Fever and dyspnoe^b^	No	++	n.d.^c^
						
N13	12.5	According to plan	ALT grade 1, GGT grade 2, AP grade 1	No	No	Progression
N14	12.5	Stopped on day 8	ALT/AST grade 3, GGT grade 2, LDH grade 1	Yes	n.d.	n.d.
N15	12.5	According to plan	ALT grade 2, AST grade 1, AP grade 2	No	+	Stable disease
N17	12.5	According to plan	ALT/AST grade 2	No	No	Progression
U04	12.5	According to plan	Dyspnoe	No	No	n.d.^c^
U05	12.5	According to plan	None	No	++	Progression
						
N09	20	According to plan	ALT/AST grade 2	No	+++	Progression
N10	20	Stopped on day 8	ALT grade 4, AST grade 3, GGT grade 2	Yes	n.d.	n.d.
N12	20	Stopped on day 8	ALT grade 3, AST grade 2	Yes	n.d.	n.d.^c^

ALT, alanine aminotransferase; AST, aspartate aminotransferase; GGT, gamma-glutamyl transferase; n.d., not determined.

^a^Causal relationship with study drug unlikely.

^b^Patient U03 developed fever and dyspnoe after therapy on day 23, which was resolved with antibiotics; the patient died on day 40, causal relationship with study drug unlikely.

^c^Clinical signs of activity while on therapy including healing of cutaneous lesion (U03, U04), size reduction of lymph node metastasis (U03), and inflammatory response and softening of large tumor mass (N12).

**Table 3:** Pharmacokinetics of scFv(FRP5)-ETA

Dose level (μg/kg)	Peak concentration range (ng/ml)	Peak concentration mean (ng/ml)	AUC (ng* hour/ml)	Plasma clearance (l/hour)	Half-life (hours)
2	n.d.	n.d.	n.d.	n.d.	n.d.
4	18–49	39 ± 18	39 ± 1	6.6 ± 0.6	0.55 ± 0.02
10	128–129	129 ± 1	138 ± 64	5.3 ± 2.1	0.74 ± 0.27
12.5	93–204	160 ± 35	178 ± 100	4.9 ± 3.1	0.73 ± 0.30
20	115–307	236 ± 105	326 ± 146	3.8 ± 2.2	0.73 ± 0.22

AUC, area under the concentration–time curve; n.d., not determined.
